# Identifying wakeup stroke routine treatments in the emergency departments

**DOI:** 10.3389/fstro.2026.1814085

**Published:** 2026-04-13

**Authors:** Mehari Gebreyohanns, Sidarrth Prasad, Kim D. Barker, Joshua D. E. Amos, Erica M. Jones, Lindsay M. Riskey, Daiwai M. Olson, Asmiet K. Techan, Ty A. Johnson, Nneka L. Ifejika

**Affiliations:** 1 Department of Neurology, UT Southwestern Medical Center, Dallas, TX, United States; 2 Ochsner Health, New Orleans, LA, United States

**Keywords:** acute ischemic stroke, emergency department, standardized protoco, thrombolytics, wakeup

## Abstract

**Background:**

The term wake-up stroke refers to an acute ischemic stroke with an unknown time of onset, typically discovered when a patient awakens with symptoms. Wake-up strokes account for up to 25% of all acute ischemic strokes. There is limited understanding of how hospitals vary in their evaluation and treatment of these cases, and institutional protocols, imaging strategies, and therapeutic decision-making for wake-up stroke remain inconsistently defined.

**Methods:**

In this prospective observational study, we surveyed hospitals in Texas and Louisiana to assess institutional approaches to wake-up stroke care including hospital characteristics, imaging protocols, treatment pathways, and decision-making criteria for acute ischemic stroke with unknown onset time.

**Results:**

Among 54 hospitals in Louisiana (29), and Texas (25), representing 48 unique zip codes, >80% followed a standardized institutional protocol when making decisions for wake-up strokes. Additionally, 75.5% of hospitals ordered MRIs for these cases in the acute setting.

**Conclusion:**

A coordinated, systems-level approach to wake-up stroke care that integrates a standardized protocol may be valuable in promoting workflow processes.

## 1 Introduction

A wake-up stroke is defined as a stroke with unknown onset time (Zhao et al., 2025). These are often excluded from thrombolytic treatment due to the absence of a standardized approach in an emergency department (ED). Up to 25% of acute ischemic strokes are wake-up strokes (Huang et al., 2021). Wake-up strokes may go untreated due to uncertainty in the time of symptom onset, as the window for administering a thrombolytic treatment ends at 4.5 h from when symptoms began (Mac Grory et al., 2021). In recent years practitioners have adopted brain imaging techniques to select some of these patients appropriate for acute thrombolytic treatment (Zhou et al., 2025). The study focused on the decision making for thrombolysis by assessing current hospital-level practices and treatment considerations for wake-up stroke across two states in the United States.

When providers are triaging wake-up stroke, in the ED, they may deem the patient ineligible for thrombolytic therapy. Some centers may start antiplatelet therapy without

ordering additional imaging, while lower resourced centers without magnetic resonance imaging (MRI) capability may not transfer these patients to comprehensive stroke centers for MRI, thrombolysis or endovascular therapy (EVT) (Huang et al., 2021; Grotta, 2023). Due to the uncertain onset of symptoms, only about 8–27% of patients experiencing wake-up strokes receive thrombolytic therapy (Manawadu et al., 2013). MRI can be crucial in estimating the ischemic core and penumbra, guiding decisions on thrombolytic therapy for these patients (Altmann et al., 2024). Furthermore, it can be cost effective in the long run, because earlier identification and treatment of the stroke patients can lead to lower rehabilitation costs (de Aguiar Kuriki and Kitamura, 2022).

Approximately 30% of patients with wake-up stroke with negative fluid attenuated inversion recovery (FLAIR) and a visible acute ischemic lesion on diffusion weighted imaging (DWI) findings on MRI, or MRI mismatch, may be candidates for thrombolytic therapy. Both intravenous (IV) thrombolytic treatment and EVT in these patients improve functional outcomes without increasing the risk of death (Roaldsen et al., 2021; Grotta, 2023). Multimodal MRI like diffusion weighted imaging, FLAIR, perfusion weighted imaging, and susceptibility weighted imaging may provide data that extend a patient's time window for acute treatment (Zhang et al., 2021).

Implementing a protocol for wake-up stroke in both lower and higher level stroke centers could improve outcomes by ensuring appropriate MRI use and timely intervention. It extends the opportunity to offer acute treatment and improve the disability-adjusted life years for these patients who are in this setting (Roaldsen et al., 2021). This may lead to expedited stroke code activation in the ED and has the potential to improve process flow (Sanjuan et al., 2023). Different healthcare systems may implement different practices in handling wake up strokes but a standard protocol would provide standardized treatment to patients and benefit researchers by providing metrics data that they can analyze to improve quality of care.

Implementation science is defined as “the scientific study of methods to promote the systematic uptake of research findings and other evidence-based practices (EBPs) into routine practice, in order to improve the quality and effectiveness of health services.” (Eccles and Mittman, 2006; Bauer and Kirchner, 2020). Our team has developed an implementation study proposal on acute stroke management in hospital settings across Texas and Louisiana. For our upcoming grant resubmission, we are collecting preliminary data to inform us of the current practices for the treatment of wake-up stroke.

## 2 Materials and methods

For this prospective observational study, institutional review board approval was obtained from the affiliated universities to collect data using a survey-based methodology. Hospitals in Texas (TX) affiliated with the Lone Star Stroke Research Consortium and hospitals in Louisiana (LA) provided survey data from September 2024 to March 2025. The unit of analysis included both hospital-level data and individual respondents from each of the 54 participating sites. The survey was developed with input from an expert panel comprising Stroke and Physical Medicine and Rehabilitation physicians and was securely hosted on the REDCap platform. Participating hospitals were contacted primarily via email or phone to complete the survey. Data collected included hospital characteristics such as setting, county, ZIP code, stroke certification status, availability of MRI (e.g., 24 h per day), and presence of an established wake-up stroke protocol. Structural capacity was assessed by asking the number of ED and hospital beds, as well as stroke-designated beds. Annual metrics included overall hospital admissions, stroke admissions, stroke codes, and the number of patients transferred for primary stroke centers and acute stroke ready hospital (ASRH). Respondents also provided estimates of the number of patients aged 65 years and older and whether they considered wake-up stroke patients eligible for thrombolysis and ultimately whether these patients received the acute treatment (Table 1). Additionally, stroke team knowledge regarding wake-up stroke management was assessed using a Likert-scale questionnaire. No protected health information (PHI) was collected. Upon completion of data collection, the cumulative REDCap dataset was exported to a Microsoft Excel spreadsheet for coding and data cleaning. Data were then uploaded into SAS v9.4 (SAS Institute) for analysis. All continuous data are expressed as mean (standard deviation) or median (interquartile range); nominal data are expressed as frequency (percent). Models were created to explore differences in with or without a standardized wake up stroke protocol Chi-Square and Kruskal-Wallis.

**Table 1:** Eligibility evaluation process metrics.

Variables	Have standard protocol	No standard protocol	*P*-Value
**Texas**	10 (40%)	15 (60%)	
**Louisiana**	29 (100%)	0 (0.0%)	**<** **0.0001**
Hospital setting			
Urban	26 (66.7%)	7 (46.7%)	**0.4493**
Suburban	3 (7.7)	6 (30.0%)	
Rural	10 (25.6)	2 (13.3%)	
**Accreditation certified**			**0.0068**
Joint commission	19 (48.7%)	10 (66.7%)	
Det norske veritas	2 (5.1%)	4 (26.7%)	
Not certified or not answered	18 (46.2%)	1 (6.7%)	
**Percentage with 24-hour radiologist coverage**	39 (100.0%)	15 (100.0%)	
**24-hour MRI capability**	39 (100.0%)	15 (100.0%)	
Hospitals considering wake-up strokes as potentially eligible for IV thrombolytic (alteplase or tenecteplase)			
No	4 (10.3%)	6 (40.0%)	**<** **0.0001**
Yes	35 (89.7%)	4 (26.7%)	
Not sure	0	5 (33.3%)	
**Acute MRI ordered for all stroke patients with unknown LKW**			**0.0062**
No	5 (13.2%)	7 (46.7%)	
Yes	33 (86.8%)	7 (46.7%)	
Not sure	0 (0.0%)	1 (6.7%)	
Missing	1	0	
**Different approach for wakeup strokes compared to unknown LKW**			**0.0035**
No	33 (84.6%)	8 (57.1%)	
Yes	5 (12.8%)	1 (7.1%)	
Not sure	1 (2.6%)	5 (35.7%)	
Missing	0	1	
**Percentage receiving thrombolysis of all patients presenting with unknown LKW**			**0.6399**
< 25%	33 (94.3%)	15 (100.0%)	
25%−50%	1 (2.9%)	0 (0.0%)	
> 50%	1 (2.9%)	0 (0.0%)	
Missing	4	0	
**Percentage receiving endovascular intervention of all patients presenting with unknown LKW**			**0.2069**
< 25%	35 (89.7%)	9 (69.2%)	
25%−50%	3 (7.7%)	3 (23.1%)	
> 50%	1 (2.6%)	1 (7.7%)	
Missing	0	2	
**Unknown LKW transfer percentage to higher level centers**			**0.5160**
< 25%	24 (70.6%)	10 (83.3%)	
25%−50%	3 (8.8%)	0 (0.0%)	
> 50%	7 (20.6%)	2 (16.7%)	
Missing	5	3	
**Percentage with regular leadership meetings to discuss stroke treatment quality improvement measures**			
Have meetings	10	14	
Missing	29	1	

Bold value indicates statistically significant difference.

## 3 Results

Of the 55 hospitals that responded to the survey, one did not complete any questions and was excluded from the analysis. The remaining 54 hospitals included 29 from LA and 25 from TX, representing 48 unique ZIP codes. Based on hospital setting, 33 (61.1%) hospitals were urban, 9 (16.7%) suburban, and 12 (22.2%) rural. All hospitals in LA were part of a single health system and followed a standardized wake-up stroke protocol. In contrast, among the TX hospitals, 10 (40.0%) reported having a wake-up stroke protocol in place, while 15 (60.0%) did not. Among the 54 hospitals included in the analysis, the mean (SD) number of total hospital beds was 303.4 (270.37), ED beds 43.8 (30.24), and dedicated stroke beds 36.4 (102.12). Annual stroke-related volumes included a mean of 570.7 (880.58) annual stroke codes and 362.7 (441.59) stroke admissions. The mean percentage of patients aged ≥65 years was 52.4 (14.34) (Table 2). Regarding certification, 29 (53.7%) were Joint commission certified, 6 (11.1%) were Det Norske Veritas (DNV) certified and 19 (35.2%) were not applicable or did not report certification. Stroke center accreditation included 17 (31.5%) Comprehensive Stroke Centers (CSC), 2(3.7%) Thrombectomy-Capable Stroke Centers (TSC), 15 (27.8%) Primary Stroke Centers (PSC), 2 (3.7%) Acute Stroke Ready Hospitals (ASRH), 7 (13.0%) state-certified centers, and 11 (20.4%) did not report their certification. All hospitals surveyed reported that they were able to obtain an MRI 24-h per day and that they had an on-duty radiologist available to interpret the MRI. All sites also confirmed that they held regular leadership meetings focused on improving stroke care at their facility. There were 40 sites which reported that they order an MRI for all stroke patients with unknown last known well (LKW), 33 of which reported having a standard protocol. For wake-up stroke management, 36 (83.7%) hospitals reported that treatment decisions were not dependent on the individual treating physician, and they followed a standardized wake up stroke protocol. When asked whether their facility generally considers wake-up stroke patients as eligible for acute stroke interventions, 39 (72.2%) indicated thrombolytic therapy and 53 (100%) indicated endovascular thrombectomy (EVT) as possible treatment options for this patient group. Among the hospitals surveyed, 48 (96.0%) reported that fewer than 25% of stroke code–activated wake-up stroke patients received thrombolytics, and 44 (84.6%) reported that fewer than 25% received EVT. When asked to assess their stroke team's knowledge in managing wake-up stroke using a Likert-scale questionnaire, 44 (83.0%) hospitals rated it as excellent, 8 (15.1%) as somewhat good, and 1 (1.9%) as neutral.

**Table 2:** Hospital characteristics for hospitals with versus without a wakeup stroke protocol.

Variables	Have protocol	No protocol	*P*-Value
Number of total admissions per year	9916.8 (10774.46)	89075.9 (176,226.72)	**0.0048**
Number of hospital beds in facility	258.2 (266.83)	421.1 (250.90)	**0.0175**
Number of annual stroke codes	366.6 (455.00)	1,120 (1,414.95)	**0.0040**
Number of stroke codes in individuals over age of 65	52.1 (13.69)	53.2 (16.77)	**0.4590**
Number of stroke admissions per year	271.4 (384.48)	659.4 (500.12)	**0.0072**
Number of stroke beds in hospital	35.1 (114.75)	41.0 (36.13)	**0.0092**
Number of ED beds in hospital	44.0 (31.59)	43.3 (27.44)	**0.8167**
Mean knowledge level of stroke providers on treatment for wake-up stroke	4.9 (0.27)	4.5 (0.65)	**0.0025**

^*^All values in parenthesis equals standard deviation. Bold value indicates statistically significant difference.

## 4 Discussion

The Wake-up stroke study provides important insights into the variability of wake-up stroke management across hospitals in Texas and Louisiana, highlighting key differences between institutions with standardized protocols and those without. Hospital characteristics such as certification status, urban vs. rural location, and stroke care volume are critical factors associated with stroke care and should be further investigated. Hospitals that reported having a standard wake-up stroke protocol tended to be part of larger healthcare systems and were more likely to have comprehensive stroke center certification, suggesting that institutional resources may influence protocol adoption. Despite universal access to MRI and 24 h radiologist coverage among the surveyed hospitals, treatment rates for wake-up stroke remained low across all settings. These findings highlight the need to explore and question whether having a protocol is associated with improved treatment rates and identify barriers that prevent protocol implementation. Additionally, prior studies have demonstrated that timely treatment of wake-up stroke patients, particularly with thrombolysis, can lead to improved functional outcomes, reinforcing the importance of standardized care pathways. Although wake up strokes account for 1 in 5 of all reported ischemic stroke patients, only 8% of these patients are treated with thrombolysis, with significantly fewer receiving EVT (Søyland et al., 2024; Zhao et al., 2025). Only a minority of hospitals surveyed reported administering thrombolytics or EVT to more than 25% of eligible wake-up stroke patients, consistent with norms surrounding treatment for this population. This suggests that the standardized treatment, or lack thereof, for patients waking up with stroke symptoms potentially poses the most significant cause to barriers in protocol application, triage behavior, and institutional readiness. Previous research has found a positive effect on functional outcomes at as well as no significant difference in mortality rates among wake-up stroke patients and patients with known stroke onset times when reassessed 3 months post-stroke (Liu et al., 2022; Kamogawa et al., 2024). Additionally, 61% of patients achieved a Modified Rankin scale score between 0 and 2, highlighting better functional outcomes (Mac Grory et al., 2021). Although it is recommended that wake up stroke patients receiving thrombolysis should still be monitored for intracranial hemorrhage transformation, other findings have shown no increased risk compared to known stroke onset patients receiving TNK for hemorrhage (Søyland et al., 2024). While hospitals differ significantly in their adoption of standardized wake-up stroke protocols, a consistent pattern persists across all settings: wake-up strokes are infrequently treated with thrombolysis. This uniform hesitancy highlights a critical gap in current practice and reinforces the need for future research to clarify the benefits, risks, and implications of maintaining—or revising— this longstanding treatment approach.

## 5 Limitations

Potential limitations within this study were having the majority of surveyed centers belong to large systems including all Louisiana centers from a single Louisiana hospital system. We did not collect data analyzing similarities and differences of the wake-up stroke protocols which would also be helpful to analyze differences in treatments outcomes. We also did not fully investigate the outcomes between centers with and without a standardized protocol. Another notable limitation of this study is the exclusion of non-wake up stroke treatment protocol questioning within the survey which would improve understanding of overall acute stroke management and efficiency.

## 6 Conclusion

The findings suggest that institutional readiness alone may not be sufficient to ensure effective implementation; rather, a coordinated approach involving protocol standardization, enhanced provider education, and system-level workflow integration is essential. Particularly in states like Texas, where protocol variability is high, implementing a unified strategy could improve treatment equity and outcomes for wake-up stroke patients. Future research should explore effectiveness of existing protocols, assess provider perspectives on their utility, and compare obstacles to wake-up stroke management with broader stroke care practices. Ultimately, advancing implementation science in this domain holds the potential to transform emergency stroke response and reduce disability and mortality associated with delayed treatment.

## Data Availability

The original contributions presented in the study are included in the article/supplementary material, further inquiries can be directed to the corresponding author.
